# Improving Performance of Quasi-2D Perovskite Light-Emitting Diodes by Solvent Atmospheric Post-Treatment

**DOI:** 10.1021/acsami.5c08632

**Published:** 2025-07-09

**Authors:** Peiyuan Pang, Ge Zeng, Yulin Mao, Bingzhe Wang, Zhipeng Zhang, Jinfeng Liao, Xiangfeng Deng, Jiangshan Chen, Dongge Ma, Guichuan Xing

**Affiliations:** † Joint Key Laboratory of the Ministry of Education, Institute of Applied Physics and Materials Engineering, 59193 University of Macau, Macau 999078, China; ‡ Institute of Polymer Optoelectronic Materials and Devices, State Key Laboratory of Luminescent Materials and Devices, Guangdong Provincial Key Laboratory of Luminescence from Molecular Aggregates, 26467 South China University of Technology, Guangzhou 510640, China

**Keywords:** quasi-2D perovskite, light-emitting diode, post-treatment, solvent atmosphere, phase distribution

## Abstract

Perovskite light-emitting diodes (PeLEDs) have achieved remarkable progress in recent years, with green-emitting PeLEDs now exhibiting external quantum efficiencies (EQEs) exceeding 30%, rivaling those of organic light-emitting diodes (OLEDs). While quasi-2D perovskite structures have emerged as a promising strategy for high-efficiency PeLEDs—owing to their enhanced exciton binding energies and charge carrier confinement—their phase distribution remains a critical yet challenging factor governing device performance. In this study, we demonstrate that the phase distribution in quasi-2D perovskite films is highly sensitive to the solvent atmosphere during annealing. Polar solvents (e.g., dimethylformamide, dimethyl sulfoxide) introduce nonradiative recombination centers and accelerate film decomposition, whereas nonpolar solvents (e.g., chlorobenzene) suppress degradation and promote high-*n*-phase purity. By employing nonpolar solvent vapor post-treatment, we achieve enhanced radiative recombination efficiency, yielding PeLEDs with a maximum EQE of 24.27%. Our findings highlight a previously overlooked aspect of perovskite film fabrication and provide key insights for the scalable production of quasi-2D PeLEDs.

## Graphical abstract

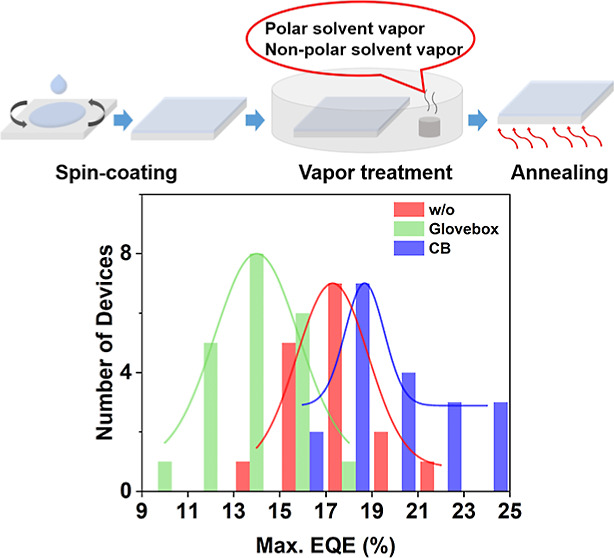

## Introduction

Metal halide perovskites (MHPs) have emerged as highly promising materials for light-emitting diodes (LEDs) in recent years owing to their exceptional optoelectronic properties. These include high photoluminescence quantum yields (PLQYs), narrow emission bandwidths, tunable band gaps, high charge-carrier mobilities, solution processability, and low fabrication costs—attributes that make them ideal candidates for next-generation lighting and display technologies.^1–8^ Recent advances in perovskite LEDs (PeLEDs) have been particularly striking, with reported external quantum efficiencies (EQEs) consistently exceeding 20% ^9–31^ and state-of-the-art devices achieving EQEs above 30%.^32–34^ A key strategy for enhancing PeLED performance involves the use of quasi-2D perovskite structures where metal-halide octahedral multilayers are separated by large organic cation spacers. Such architectures significantly improve the PLQY by increasing exciton binding energies and confining charge carriers. However, solution-processed quasi-2D perovskite films typically comprise mixed multiple quantum wells (MQWs) with varying well widths (i.e., differing numbers of octahedral layers). The presence of low-dimensional phases—particularly those with small *n*-values (few octahedral layers)—severely limits device efficiency due to strong exciton–phonon quenching at room temperature.^35–37^ Consequently, precise phase control and regulation are critical for developing high-efficiency quasi-2D PeLEDs.^38–42^

Due to the small Gibbs free energy differences among multiple *n*-phases and the rapid nucleation kinetics, obtaining a pure quasi-2D perovskite phase with strict stoichiometric ratios via solution processing remains challenging.^43–46^ In quasi-2D perovskites with mixed *n*-phases, exciton energy transfers from low-*n* phases (wide band gap) to high-*n* phases (narrow band gap). This carrier funneling effect significantly enhances radiative recombination under low excitation intensities—provided a smooth energy funnel is constructed.^47,48^ However, excessive low-*n* phases, which hinder energy transfer and radiative recombination, tend to dominate crystallization due to their lower formation energy.^44^ To achieve an optimized energy funnel, strategies such as tailored spacer cation design,^23,36^ mixed spacer cations,^49,50^ additive engineering,^35,40,51^ and post-treatment^52,53^ have been explored. Notably, the role of solvent atmosphere—often overlooked—is critical during solution-phase perovskite film fabrication. Polar solvents dissolve perovskite precursors, while nonpolar antisolvents assist crystallization. Their vapors constitute the primary solvent atmosphere during film formation. Recent advances in PeLED fabrication have employed vapor treatments: polar solvent vapors induce recrystallization, ion redistribution, and ion exchange, whereas nonpolar vapors primarily facilitate annealing.^54–59,67^ Even without intentional vapor treatment, the solvent atmosphere inevitably influences film properties, particularly in batch processing. Inconsistent control of the processing parameters (e.g., solvent vapor concentration and exposure duration between successive batches) often compromises device reproducibility. Despite its significance, systematic studies of solvent atmosphere effects remain scarce.

Here, we demonstrate that quasi-2D perovskite films show significantly different final phase distributions when post-treated with different solvent atmosphere before annealing. Polar vapor will trigger the recrystallization of perovskite films, and low-dimensional phases are preferred to form, which is not conducive to radiative recombination. The solvent vapor with a stronger polarity will even decompose the perovskite structure. Therefore, in the process of preparing perovskite thin films, the contact between perovskite films and the polar vapor should be avoided as far as possible. Even a small amount of polar steam in the nitrogen glovebox can significantly change the morphology of perovskite films and reduce PLQY. In view of this, we propose a vapor treatment method in which the prefabricated perovskite films are treated in a saturated nonpolar vapor atmosphere for a period of time prior to annealing. On the one hand, the perovskite films are protected from the negative effects of polar vapors, and second, during the nonpolar vapor treatment process, the residual polar solvents in perovskite films are extracted. In this case, the crystal undergoes a slow growth process, which can effectively promote the growth of dense, large grains compared with the rapid crystallization of direct annealing. This slow growth process improves the reproducibility of film batches and increases the radiative recombination efficiency of the films.

## Results and Discussion

The precursor solution is prepared by dissolving cesium bromide (CsBr), lead bromide (PbBr_2_), phenylethylammonium bromide (PEABr), and sodium bromide (NaBr) in dimethyl sulfoxide (DMSO). PEABr is introduced as spacer material and combined with excessive CsBr (CsBr/PbBr_2_ = 1.6:1) to construct quasi-2D/CsPbBr_3_/Cs_4_PbBr_6_ nanocomposite emissive layer (EML). The Cs_4_PbBr_6_ with a large band gap can centralize carriers for radiative recombination and confine excitons for efficient energy transfer, as reported in our previous work.^60^ For the control sample labeled as w/o, the precursor solution is spin-coated onto substrates for 90 s and then immediately transferred to the hot plate for annealing. For the samples prepared to study the effects of different atmospheres on the films, after spin-coating, the films are exposed to different atmospheres for 5 min, and then transferred to the hot plate for annealing (Figure 1a). Four atmospheres of original glovebox atmosphere (glovebox), glovebox atmosphere saturated with chlorobenzene (CB), glovebox atmosphere saturated with dimethylformamide (DMF), and glovebox atmosphere saturated with DMSO are selected for comparison.

**Figure 1:**
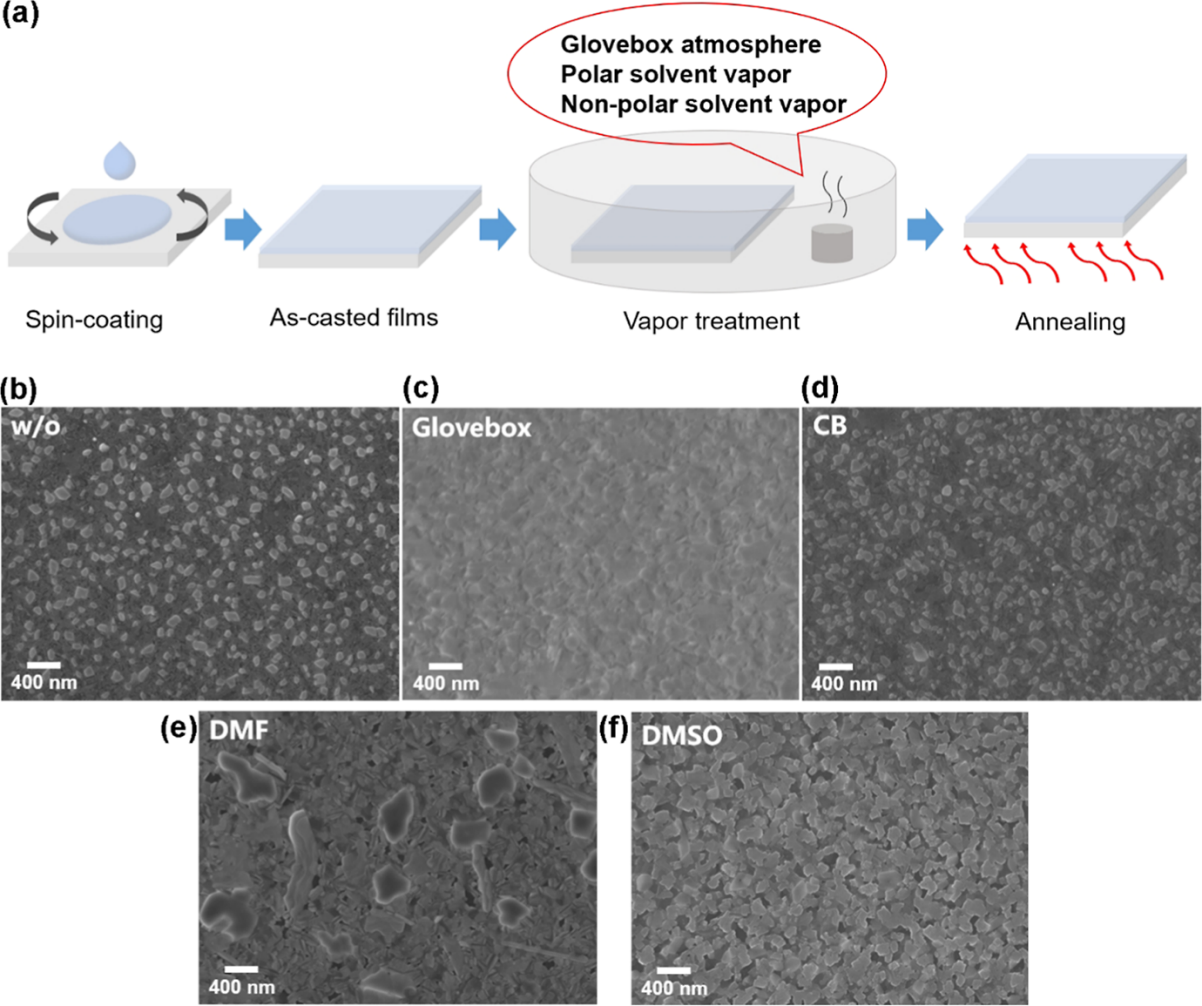
Fabrication and morphological characterization of perovskite films treated under different atmospheres. (a) Fabrication procedure of perovskite films. (b–f) SEM images of perovskite films treated under different atmospheres.

Both the w/o and CB-treated samples display similar morphological features, consisting of dispersed bulk grains distributed across layered-phase matrices (Figure 1b,d). This morphology suggests a mixed-phase composition containing 3D, quasi-2D, and potentially 0D perovskite phases. The spatially confined bulk grains are particularly crucial for enabling efficient radiative recombination, ultimately enhancing the performance of the resulting PeLED devices. Notably, the small crystalline particles observed in the w/o sample, which are dispersed within the layered quasi-2D phase (as marked in Figure S1a), are primarily products of rapid crystallization. In contrast, the CB-treated sample exhibits only a minimal amount of such small particles, instead displaying a dominant composite structure of dense bulk phase and layered phase, characteristic features of a slow crystal growth process (Figure S1b). For the Glovebox sample, bulk and layered phases can still be observed on the surface, but their boundaries become blurred (Figures 1c and S1c). This morphology reflects the tendency of phase decomposition and recrystallization, which may be caused by the small amount of polar solvents in the glovebox. Under the influence of polar solvent vapor, the morphology of the films changes obviously. The films treated with DMF vapor contain more strip-shaped low-dimensional phases and large 3D phases (Figure 1e). This may be due to the recrystallization process resulting in the growth of low-dimensional and high-dimensional phases. However, the films treated with DMSO vapor contain a large number of stacked strips and bulk phases, which are the products of complete recrystallization (Figure 1f). It is worth noting that in the low-magnification images, large massive structures appear on the surface of DMSO sample (Figure S2a). According to EDS analysis (Figure S2b,d), the major component of these massive structures can be inferred to be lead bromide, indicating that the films were decomposed under the influence of strong polar solvent vapor DMSO.

For quasi-2D perovskites, changes in the morphology often imply alterations in phase distribution, which strongly affects the radiative recombination efficiency and ultimately determines the performance of the device. We then carried out ultraviolet–visible (UV–vis) absorption and photoluminescence (PL) spectra to investigate the phase distribution. The perovskite films treated with DMF and DMSO vapor show two low-dimensional absorption peaks located at 402 and 435 nm, corresponding to *n* = 1 and *n* = 2 phases, respectively, while other films do not show significant low-dimensional absorption peaks (Figure 2a). X-ray diffraction (XRD) analysis also confirms the formation of *n* = 1 phase (Figure 2c).^61–64^ In addition, the absorption edges of these two samples showed more pronounced exciton peaks than other samples (as shown in the partially enlarged Figure 2a), indicating an increase in the 3D phase, consistent with the SEM images and the red-shifted PL spectra (Figure 2b). This is because the polar solvent dissolves a portion of the perovskite to form a DMSO or DMF coordinated precursor, which is recrystallized during the annealing. In this process, part of the dissociative precursor preferentially forms the low-dimensional phase with lower formation energy,^44^ while the other part of the precursor attached to the high-dimensional phase promotes the further growth to 3D phase in the form of secondary nucleations.^65^ Due to the difference in molecular formulas between 0D, 3D and quasi-2D perovskite structures, we can compare the ratios of Cs/Pb to prove the trend of phase distribution changes. X-ray photoelectron spectroscopy (XPS) measurements are performed to analyze the Cs/Pb ratio in the films (Figure 2d). The Cs/Pb ratios of w/o, CB, and glovebox samples are all greater than 1, indicating that the Cs_4_PbBr_6_ phase and CsPbBr_3_ phases are the main phases in the films, and the proportion of quasi-2D phases is low. The Cs/Pb values of DMF and DMSO samples are 0.96 and 0.64, respectively, indicating that the proportion of quasi-2D phases in the film increases, along with the decrease of the Cs_4_PbBr_6_ phase. Although the w/o and CB samples are very similar in morphology, the absorption edge of the CB sample is sharper, corresponding to more pure 3D phase, which corresponds to the dense bulk phases in the SEM image. While the band edge of w/o sample shows lower slope, corresponding to a series of high-*n* phases, which corresponds to the small particle phases of different sizes in SEM images. We speculate that compared with the rapid crystallization of direct annealing, CB vapor treated films undergo a slow crystallization process, which is conducive to the formation of dense large-sized grains, while avoiding the low-dimensional phase caused by recrystallization.

**Figure 2:**
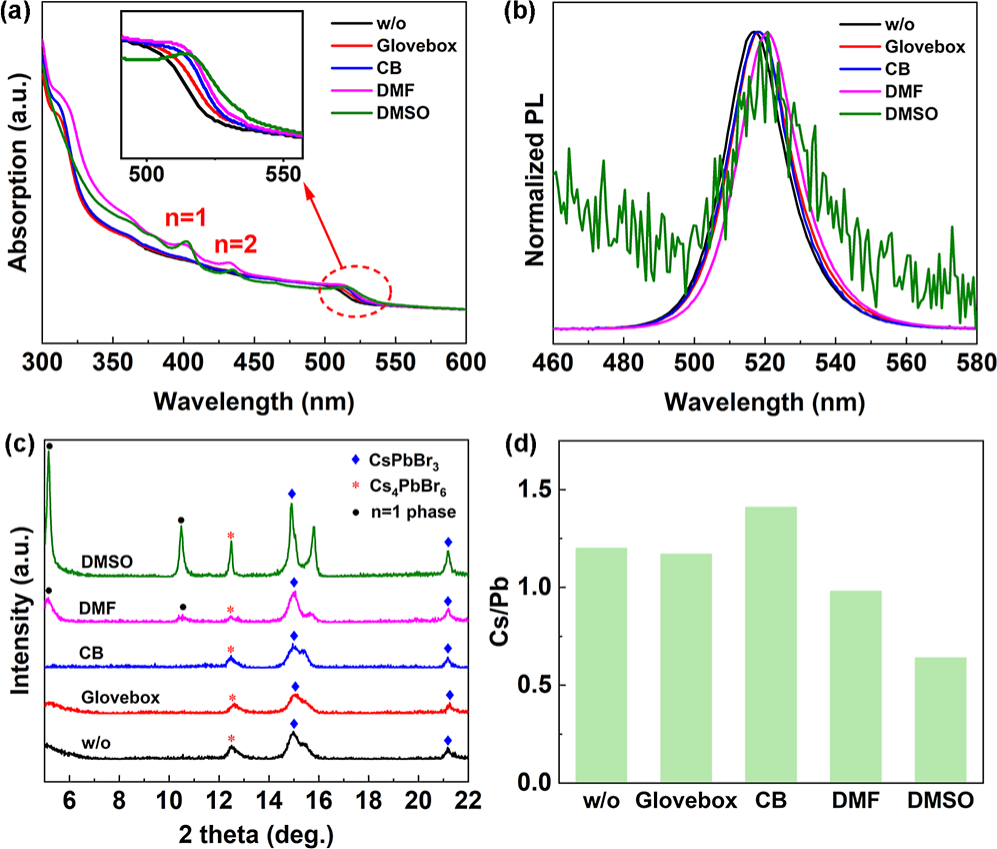
Optical, crystallinity, and component properties of perovskite films. (a) Absorption and (b) PL spectra. (c) XRD patterns of perovskite films on PVK substrates. (d) Cs/Pb elemental ratio analysis for perovskite films processed under varying solvent atmospheres.

We further probed the exciton energy transfer dynamics and phase distribution through transient absorption (TA) spectroscopy. Both the w/o and CB-treated samples exhibit single bleaching peaks at 513 and 515 nm, respectively (Figure 3a,b), suggesting dominant populations of high-*n* phases and 3D phases in these films. It is worth noting that a small amount of the *n* = 3 phase (bleaching peak located at 462 nm) can be found in the glovebox sample (Figure 3c), which may be induced by the residual polar solvent in the glovebox atmosphere. For films treated with DMF and DMSO vapors, distinct bleaching peaks associated with low-*n* phases emerge (Figure 3d,e). The more polar DMSO vapor promotes the formation of a greater proportion of quasi-2D phases compared with DMF. These observations align well with the steady-state absorption spectra (Figure 2a), confirming the consistent phase distribution trends across both measurement techniques. The TA traces of the ground-state bleaching (GSB) peaks are extracted to analyze the carrier transfer process. The evolution of GSB peaks can be fitted by a multiexponential function (Figure 3f), and the fitting results are summarized in Table S1. The fast τ_1_ is related to the build-up process while the other τ_2_–τ_4_ are related to the complex decay processes. The CB sample shows a relatively shorter build-up time, indicating a faster carrier accumulation to the emissive recombination centers, while the DMF sample shows significant longer build-up time. This is due to the uneven phase distribution (mainly dominated by *n* = 2 and 3D phases) usually leading to retarded carrier accumulation and inefficient energy funneling. In contrast, the build-up times of the w/o and Glovebox samples with purer phase are close to that of CB sample. It is worth noting that a three-exponential function is used to fit the TA data of the DMSO sample, indicating that the carrier dynamics are different. Its build-up time is the shortest due to the wider distribution of quasi-2D phases, building a smooth energy transfer channel. Despite having a faster carrier accumulation, the intensity of GSB peak is the weakest compared to other samples, which is also reflected in its shorter τ_2_ and τ_3_. This is because the decomposition of the film leads to a large reduction in the radiative recombination center, which is accompanied by severe fluorescence quenching.

**Figure 3:**
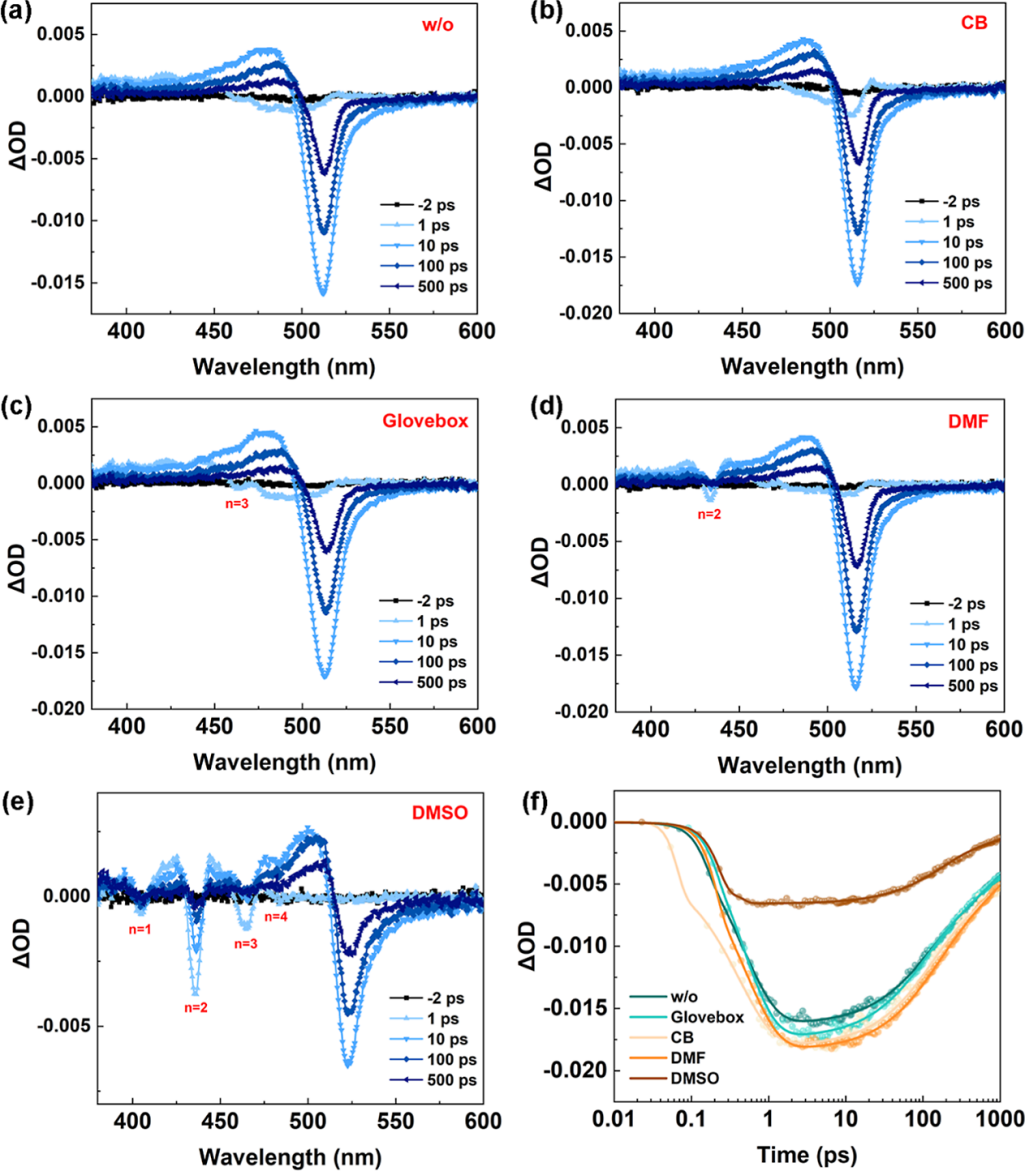
TA characterizations of the perovskite films. (a–e) TA spectra at different probe delay times for the samples of w/o, CB, glovebox, DMF, and DMSO, respectively. (f) TA kinetics at each GSB peaks for the samples of w/o, CB, glovebox, DMF, and DMSO, respectively. Following excitation at 360 nm (100 fs, 1 kHz, 1.6 μJ/cm^2^).

To achieve highly efficient quasi-2D perovskite, efficient energy transfer and fewer nonradiative recombination centers are necessary. Both w/o and CB samples exhibit a high PLQY of nearly 70% (Figure 4a) based on their efficient energy transfer processes. In addition, we introduced cyclohexane (Cy) and toluene (Tol) as two nonpolar vapor samples for comparison, their PLQYs and morphologies are close to that of CB sample, proving that different nonpolar vapor has similar effects on quasi-2D perovskite (Figure S3). On the other hand, the PLQYs of the films after polar vapor treatment decrease significantly, in which the DMF sample is 30.7%, and the DMSO sample with stronger polarity is 2.4%, which is consistent with the previous SEM and TA analysis results. It is worth noting that the PLQY of the glovebox sample also decreased significantly (41.6%). Combined with the analysis results of morphology and phase distribution, it can be seen that a small amount of polar solvent remaining in the glovebox has a very obvious negative effect on the films. We then conducted time-resolved photoluminescence (TRPL) measurements to investigate the underlying mechanism. The TRPL curves are fitted by a triexponential function (Figure 4b), and the corresponding data are listed in Table S2. The lifetimes τ_1_, τ_2_, and τ_3_ represent the fast, intermediate, and slow decay components, respectively. According to previous work,^35^ the slow component can be attributed to the radiative recombination, whereas the fast and intermediate components can be attributed to two different trap-assisted recombination. Combined with τ_avg_ and PLQY, we calculate the radiative and nonradiative recombination rate (*k*_rad_, *k*_nonrad_) of the films. First, w/o and CB samples with similar PLQY are compared, and the *k*_rad_ of CB sample is higher because the more pure high-dimensional phases assisted by CB vapor are conducive to radiative recombination. Polar vapor samples (DMF and DMSO) exhibit lower *k*_rad_ and higher *k*_nonrad_. The *k*_nonrad_ of these two samples is of the same order of magnitude because the nonradiative recombination is dominated by the low-dimensional phases,^35,66^ and the *k*_nonrad_ increases with the increase of the low-dimensional phases. However, the *k*_rad_ of DMSO sample is 1 order of magnitude smaller than that of DMF sample because the decrease of *k*_rad_ is affected by the degradation of the film here, and the stronger polarity of DMSO makes the decomposition of the film more serious, resulting in severe fluorescence quenching. For the glovebox sample, *k*_nonrad_ is close to the DMF sample, indicating that even a small amount of low-dimensional phases will lead to significant nonradiative recombination, and the decrease of *k*_rad_ also indicates that the film has a partial degradation, which is consistent with the SEM results. A schematic diagram of the crystallization process of as-casted perovskite films under polar, nonpolar vapor treatment, and without treatment is shown in Figure 4c. Most of the DMSO-coordinated precursors react during the spin-coating process but a few remain. After direct annealing without any treatment, DMSO is rapidly evaporated, and the remaining part remains in the form of the amorphous phase. Due to the rapid crystallization process, the films tend to form a series of grains of different sizes. In the nonpolar vapor atmosphere, the DMSO in the films is slowly extracted and volatilized, while the residual part of the precursor attached to the high-dimensional phase promotes the further growth to 3D phase in the form of secondary nucleations. This slow crystallization process significantly increases the grain size in the film, and the 3D phase is purer. DMF or DMSO can penetrate and partially dissolve the crystallized perovskite, forming precursor complexes. These precursors require additional consumption of PEABr or CsBr to reconvert into perovskite phases. Consequently, they extract components from existing perovskite phases to form new grains, predominantly low-dimensional phases with lower formation energy.^65^ The extraction process removes most of the PEABr and a small amount of CsBr from the original perovskite phase. The remaining CsBr and PbB_2_ components then promote further growth in the 3D phase through a secondary nucleation mechanism. This recrystallization process produces more low-dimensional and 3D phases, along with perovskite phase which decomposes into a mass of amorphous phases.

**Figure 4:**
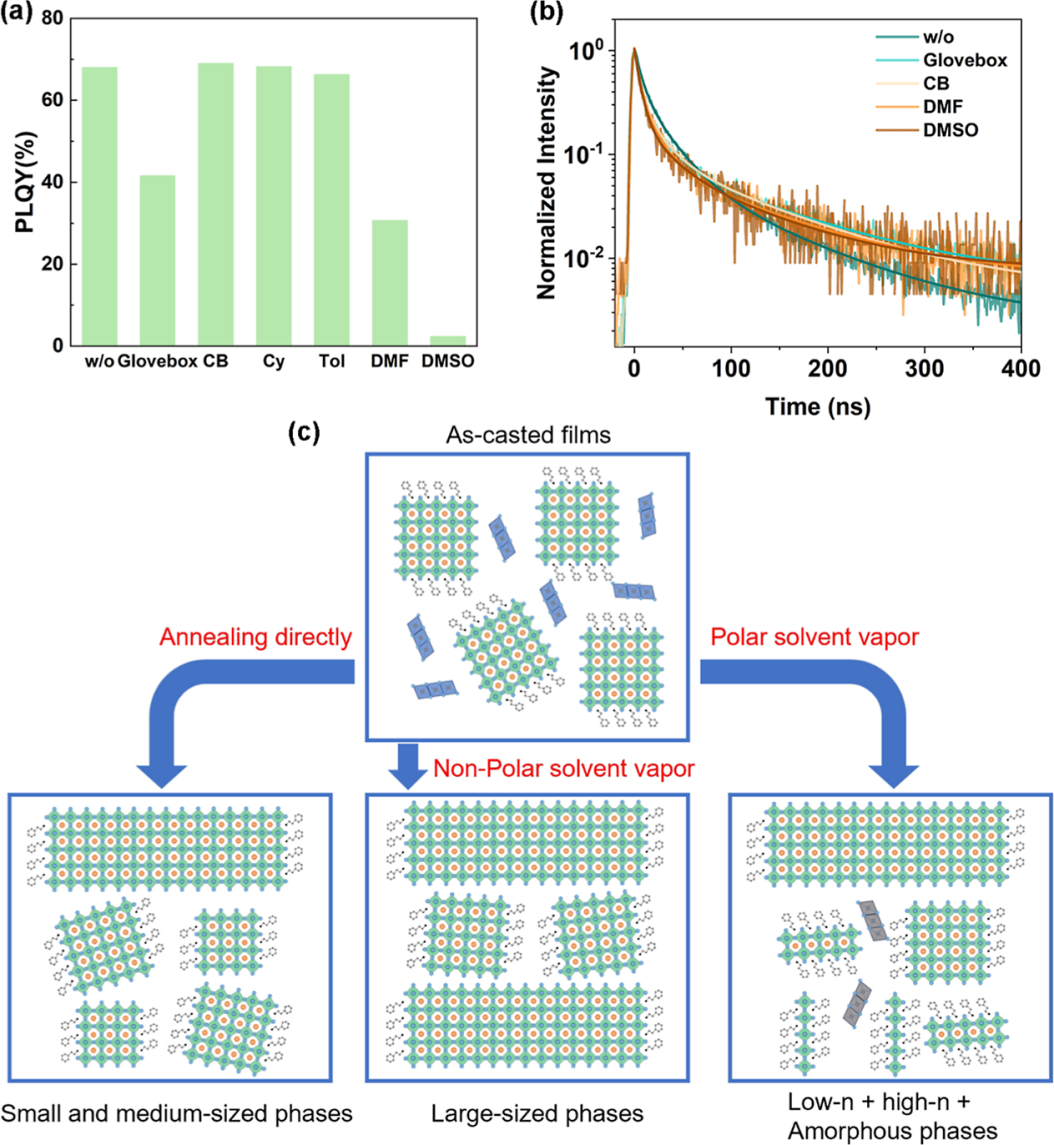
Understanding the effect of different atmospheres on the crystallization of quasi-2D perovskites. (a) PLQYs excited with 365 nm LED and (b) PL decay at the emission peaks excited with 360 nm laser pulses (100 fs, 1 kHz, 1.6 μJ/cm^2^) for the samples of w/o, CB, glovebox, DMF, and DMSO, respectively. (c) Schematic of the effect of different atmospheres on the crystallization process.

Based on the above results, we fabricate the PeLEDs with perovskite films treated with nonpolar vapor atmosphere (CB), polar vapor atmosphere (DMF and DMSO), glovebox atmosphere (glovebox), and without any treatment (w/o) as EML. The device structure is indium tin oxide (ITO, 190 nm)/nickel oxide (NiO_*x*_, 20 nm)/poly­[bis­(4-phenyl)­(2,4,6-trimethylphenyl)­amine]/poly­(9-vinylcarbazole) (PTAA/PVK, 15 nm)/perovskite (50 nm)/2,4,6-Tris­[3-(diphenylphosphinyl)­phenyl]-1,3,5-triazine (PO-T2T, 40 nm)/lithium fluoride (LiF, 1 nm)/aluminum­(Al, 100 nm), as illustrated in Figure 5a. Here, NiO_*x*_ is used as the hole injection layer, PTAA/PVK as the hole-transport layer, and PO-T2T as the electron transport layer. The valence band and conduction band of the perovskite emitters were obtained by combining the ultraviolet photoelectron spectroscopy (UPS) results (Figure S4) with the optical band gaps from absorption (Figure S5). Figures 5b–d and S6 present the current density–luminance–voltage (J–L–V) characteristics, external quantum efficiency versus luminance (EQE-L) dependence, and normalized EL spectra of the PeLEDs. The performance of PeLEDs is summarized in Table S3; the device without treatment shows a maximum EQE of 21.75% with an EL peak at 520 nm. As expected, with the CB vapor atmosphere treatment, the device shows an increase in EQE to 24.27%, accompanied by an increase in luminance. Although the highest EQE is close, PeLEDs with CB atmosphere treatment show a higher average EQE, and more devices with EQE > 20%. On the contrary, the EQEs of PeLEDs without treatment are more concentrated in the range of 15 ∼ 19% (Figures S7 and S8). The operational stability of the fabricated devices, measured under continuous operation, is shown in Figure S9. A series of different size grains formed by the rapid crystallization process not only affects the reproducibility of film batches but also complicates the energy transfer path and reduces the energy transfer efficiency. In contrast, the dense, large-sized grains formed under the CB atmosphere treatment ensure that different batches of films have efficient energy transfer paths, thus improving the PeLED efficiency. Furthermore, PeLEDs treated with nonpolar solvent vapors of cyclohexane (Cy) and toluene (Tol) also exhibited EQE values comparable to CB-treated PeLEDs, demonstrating the universality of the nonpolar solvent effect (Figure S10). Devices treated with polar vapor atmospheres (DMF and DMSO) show a significant decrease in the EL performance, with a maximum EQE of 5.58% and 0.04%, respectively, accompanied by a severe decrease in luminance. The quasi-2D phase separation caused by recrystallization and film decomposition is the main reason for the decrease in the device efficiency. It is worth noting that although the treatment time in the glovebox atmosphere is only 5 min, the EQE of the glovebox sample is also significantly lower than that of the w/o and CB samples. Considering that the polar vapor released from the residual precursor solution in the atmosphere is unavoidable in the process of batch preparation of perovskite films, this factor leading to performance degradation is often easily overlooked. The introduction of nonpolar vapor CB to assist crystallization and protect films from decomposition will be an effective strategy to improve the performance and reproducibility of mass-produced PeLEDs.

**Figure 5:**
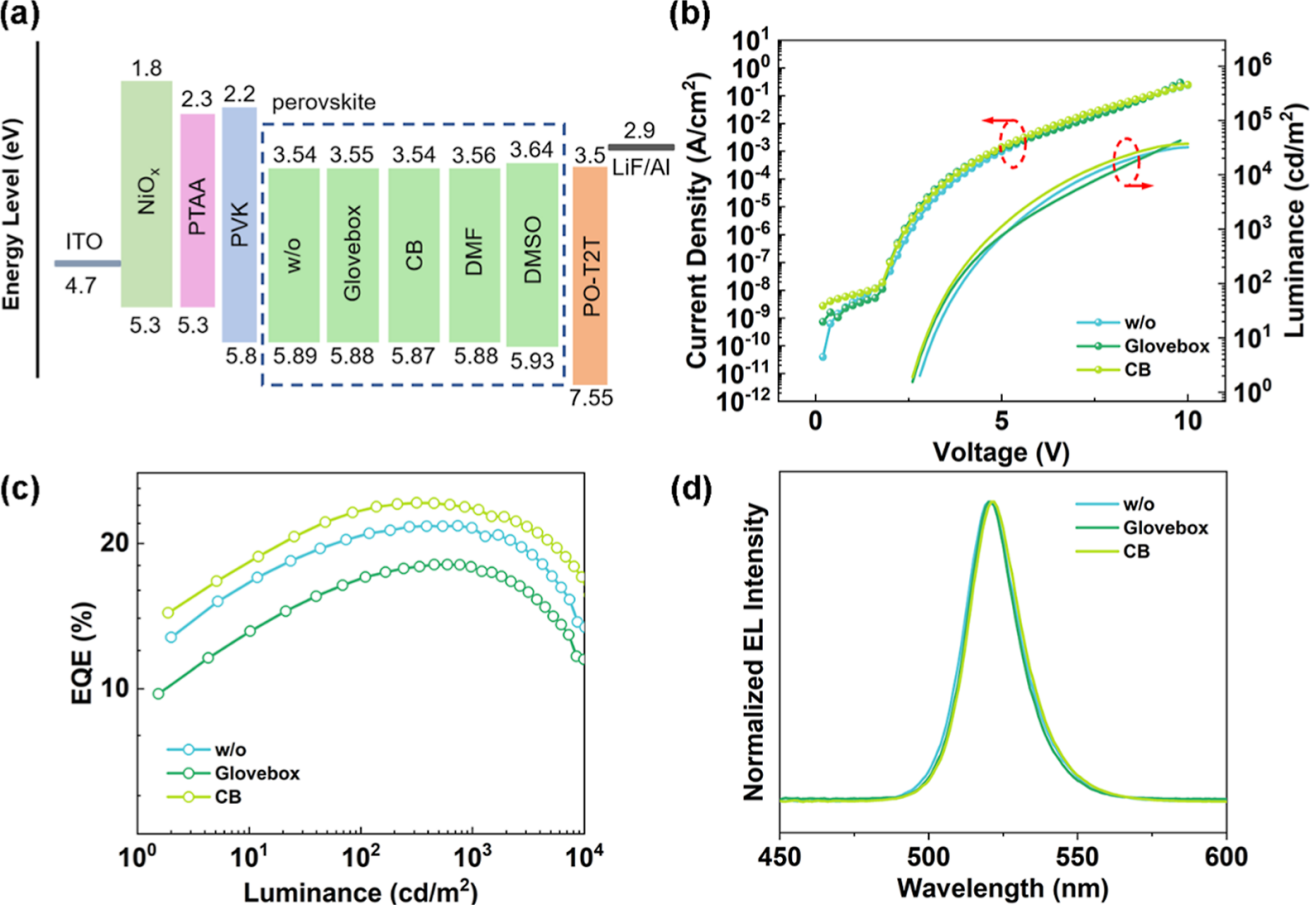
Device performances of PeLEDs treated under different atmospheres. (a) Device configuration diagram. (b) J–L–V characteristics. (c) EQE versus luminance curves. (d) EL spectra of PeLEDs measured at an applied voltage of 8 V.

## Conclusions

In summary, we have revealed that the final phase distributions of quasi-2D perovskites can be significantly affected by different solvent atmospheres before annealing. On the one hand, a polar solvent atmosphere will induce the formation of low-dimensional phases, which are unfavorable for radiative recombination, and on the other hand, it will also lead to gradual degradation of the films. Even a very small amount of the residual polar solvent will significantly impair the performance of quasi-2D perovskites. Given this circumstance, we propose a vapor treatment method by soaking the prefabricated perovskite films in a saturated nonpolar vapor atmosphere for a period of time before annealing. This method not only protects the perovskite films from decomposition and recrystallization but also promotes the formation of dense quasi-2D perovskite with large-sized grains, thus improving the radiative recombination efficiency and device reproducibility. Our study reveals an easily ignored problem in the preparation of perovskite films and proposes an effective solution, which has a guiding significance for the large-scale production of perovskite films.

## Experimental Section

### Materials

Nickel acetate tetrahydrate (Ni (CH_3_COO)_2_·4H_2_O) (99.9%) and NaBr (99.99%) were purchased from Aladdin. PEABr (99.5%) was purchased from Xi’an Polymer Light Technology Corporation. PVK (Mv = 25,000–50,000), PbBr_2_ (99.999%), CsBr (99.999%), DMSO (anhydrous, 99.9%), ethanol (anhydrous), and CB (anhydrous, 99.8%) were purchased from Sigma-Aldrich. Ethanolamine (99%) was purchased from ACROS. PTAA, PO-T2T, and LiF were purchased from Jilin OLED Photoelectric Material Corporation. All chemicals were used as received.

### Preparation of Perovskite Precursor Solutions

The perovskite precursor solution was prepared by dissolving PEABr, NaBr, CsBr, and PbBr_2_ in DMSO at a molar ratio of 0.4:0.05:1.6:1.05, respectively. The concentration of Pb was kept at 0.315 M. The solution was stirred at 45 °C overnight, and then the solution was stood for 4 h at room temperature and filtered with the polytetrafluoroethylene filter (0.22 mm) before use.

### Device Fabrication

The ITO glass substrates were ultrasonically cleaned sequentially with detergent, deionized water, and ethanol (10 min each), then dried overnight in an oven at 90 °C. The NiO_*x*_ precursor was prepared using the previous method^68^ and then spin-coated on ITO at 4000 rpm for 30 s followed by annealing at 350 °C for 60 min in air. Then, the substrates were transferred into a nitrogen-filled glovebox after cooling. PTAA was spin coated at 4000 rpm from CB solution with a concentration of 8 mg mL^–1^ and annealed at 150 °C for 30 min, then PVK was spin coated at 4000 rpm from CB solution with a concentration of 8 mg mL^–1^ and annealed at 160 °C for 30 min. Perovskite films were spin coated at 4000 rpm for 90 s. For the control samples, the films were transferred to the hot plate for annealing immediately. For the glovebox samples, the films were exposed to the glovebox atmosphere for 5 min. For the solution atmosphere treatment samples, the films were transferred to a capped Petri dish and placed for 5 min, in which 1 mL of the corresponding solvent was added in advance and placed for 15 min. The treated films were thermally annealed on a hot plate at 110 °C for 10 min. Subsequently, the devices were transferred to a vacuum deposition system for sequential thermal evaporation of the following layers: PO-T2T (40 nm), LiF (1 nm), and Al cathode (100 nm). The active device area of 5 mm^2^ was defined by the overlapping region between the Al cathode and ITO anode.

### Characterizations

UV–vis absorption spectra were acquired using a Shimadzu UV-3600 spectrophotometer. PL spectra were measured with a Horiba Fluoromax-4 spectrofluorometer (excitation wavelength: 365 nm). TRPL was recorded using a Hamamatsu streak camera system (temporal resolution: 1 ps) coupled with a Coherent Astrella-1K-F Ti:sapphire amplifier laser system (<100 fs pulse width, 1 kHz repetition rate). PL quantum yield (PLQY) measurements were performed using an Ocean Optics commercial system with 365 nm LED excitation. X-ray diffraction (XRD) patterns were collected on a Rigaku SmartLab 9 kW diffractometer using quartz substrates. X-ray photoelectron spectroscopy (XPS) measurements were conducted on a Thermo Fisher Scientific Escalab 250 Xi system. UPS was performed using a Thermo Scientific ESCALAB Xi + spectrometer. TA spectra were acquired using an Ultrafast Systems HELIOS spectrometer. The system utilized the same Coherent Astrella laser source, with 360 nm pump pulses generated by a Light Conversion TOPAS-C optical parametric amplifier. Probe pulses were produced by focusing 800 nm fundamental pulses onto a CaF_2_ plate. Scanning electron microscopy (SEM) images and energy-dispersive X-ray spectroscopy (EDS) data were obtained using a Zeiss Sigma field-emission scanning electron microscope. Film thickness was measured with a Bruker DektakXT profilometer. PeLEDs were tested on the side entrance port of the integration sphere, which collected the forward light emission in a N_2_-filled glovebox at room temperature. A fiber integrated sphere (FOIS-1) coupled to a PMA spectrometer (PMA-12, Hamamatsu) was used to characterize the emission light. The J–V curves were obtained using a source meter (Keithley 2400, Tektronix). The output data were summarized and calculated by using a computer.

## Supplementary Material



## Notes

Parameters extracted from the TA spectra of perovskite films treated under different atmospheres; transient and steady-state optical properties of perovskite films treated under different atmospheres; performance of PeLEDs treated under different atmospheres; SEM images of perovskite films without treatment and treated under CB atmospheres; morphological and component characterization of the perovskite films treated under DMSO solvent vapor; SEM images of perovskite films treated under nonpolar solvent vapor cyclohexane and toluene; high-binding energy secondary-electron cutoff and VB-edge regions of perovskite films treated under different atmospheres; optical band gaps of perovskite films extracted from absorption spectra; device performances of PeLEDs treated under DMF and DMSO atmospheres; and histogram of peak EQEs of the devices without treatment, treated under glovebox and CB atmospheres (PDF)
