# Supplementary material for: Improving Performance of Quasi-2D Perovskite Light-Emitting Diodes by Solvent Atmospheric Post-Treatment
Source: ACS Appl Mater Interfaces. 2025 Jul 9;17(29):42108–17. doi: 10.1021/acsami.5c08632 (PMC12291083; doi:10.1021/acsami.5c08632)
Supplement: Supplementary file 1 [file am5c08632_si_001.pdf]

Supporting Information

Improving Performance of Quasi-2D Perovskite Light-Emitting Diodes by Solvent Atmospheric Post-Treatment

Peiyuan Pang[1, #], Ge Zeng[1, #], Yulin Mao[1], Bingzhe Wang[1], Zhipeng Zhang[1], Jinfeng Liao[1], Xiangfeng Deng[1], Jiangshan Chen[2], Dongge Ma[2] and Guichuan Xing[1, *]

[1] *Joint Key Laboratory of the Ministry of Education, Institute of Applied Physics and Materials Engineering, University of Macau, Macau 999078, China.*

[2] *Institute of Polymer Optoelectronic Materials and Devices, State Key Laboratory of Luminescent Materials and Devices, Guangdong Provincial Key Laboratory of Luminescence from Molecular Aggregates, South China University of Technology, Guangzhou 510640, China.*

# These authors contribute equally to this work.

* Corresponding author. E-mail: gcxing@um.edu.mo

Table S1 Parameters extracted from the TA spectra of perovskite films treated under different atmospheres.

Perovskite emitter	Emission [nm]	A_1	τ_1 [ps]	A_2	τ_2 [ps]	A_3	τ_3 [ps]	A_4	τ_4 [ps]
w/o	513	0.006	0.46	-0.0031	89.90	-0.0033	587.30	-0.0017	7949
Glovebox	515	0.0067	0.43	-0.0036	83.56	-0.0032	546.41	-0.0018	7973
CB	515	0.0062	0.42	-0.004	106.24	-0.0028	694.59	-0.0022	7335
DMF	517	0.0058	0.51	-0.0037	117.92	-0.0034	659.09	-0.0020	8249
DMSO	523	0.063	0.05	-0.002	161.53	-0.0013	1614		

The four-exponential fitting reveals a rapid build-up process (τ_1) corresponding to carrier accumulation, where the subsequent decay components τ_3 and τ_4 are attributed to defect-assisted recombination and radiative recombination processes, respectively, based on their characteristic timescales. Notably, the τ_2 component displays a distinct plateau region in Figure 3f, which we interpret as a combined process involving both energy transfer (potentially originating from interactions between high-n phases, Cs_4PbBr_6 and $CsPbBr_3$) and defect-assisted recombination. In contrast, the three-exponential fitting for DMSO-treated samples—characterized by broader quasi-2D phase distribution that promotes more efficient energy transfer channels—shows three distinct timescales: τ_1 corresponding to rapid carrier accumulation, τ_2 representing defect-assisted recombination, and τ_3 associated with radiative recombination.

Table S2 Transient and steady-state optical properties of perovskite films treated under different atmospheres.

Perovskite emitter	Emission [nm]	A_1	τ_1 [ns]	A_2	τ_2 [ns]	A_3	τ_3 [ns]	τ_{avg} [ns]	QY [%]	k_{rad} [s^{-1}]	k_{nonrad} [s^{-1}]
w/o	513	0.60	7.04	0.38	26.64	0.07	100.21	20.34	68	3.3×10^7	1.6×10^7
Glovebox	515	0.67	4.96	0.26	22.03	0.09	116.73	19.17	41.6	2.2×10^7	3.0×10^7
CB	515	0.69	5.23	0.26	21.22	0.10	109.59	19.13	69	3.6×10^7	1.6×10^7
DMF	517	0.68	5.08	0.29	18.91	0.08	107.41	16.70	30.7	1.8×10^7	3.0×10^7
DMSO	523	0.78	5.71	0.17	23.33	0.08	94.38	15.51	2.4	1.5×10^6	6.3×10^7

The decay curves are fitted by the tri-exponential function:

$$I = A_1 e^{-t/\tau_1} + A_2 e^{-t/\tau_2} + A_3 e^{-t/\tau_3}$$

Where, I is the normalized photoluminescence intensity; A_1, A_2 and A_3 are the decay amplitudes; τ_1, τ_2 and τ_3 correspond to the lifetime constants of a fast component, a middle component and a slow component, respevtively. The τ_{avg} is given by the formula:

$$\tau_{avg} = \frac{A_1\tau_1 + A_2\tau_2 + A_3\tau_3}{A_1 + A_2 + A_3}$$

Since τ_{ave} could be given by

$$\frac{1}{\tau_{avg}} = k_{rad} + k_{nonrad}$$

and the PLQY could be given by

$$PLQY = \frac{k_{rad}}{k_{rad} + k_{nonrad}}$$

it follows that

$$k_{rad} = \frac{PLQY}{\tau_{avg}}$$

Table S3 Performance of PeLEDs treated under different atmospheres.

Device	V_{on} a) [V]	L_{max} [cd/m^2]	CE_{max} [cd/A]	EQE_{max} [%]	λ_{max} [nm]
w/o	2.8	32112	75.74	21.75	520
Glovebox	2.6	42761	62.93	18.07	521
CB	2.6	37009	84.50	24.27	522
DMF	3.6	474.7	21.29	5.58	522
DMSO	6.2	37.88	0.17	0.04	524

a) The turn-on voltages of PeLEDs are obtained at the luminance of 1 cd m^{-2} according to the *J-L-V* curves

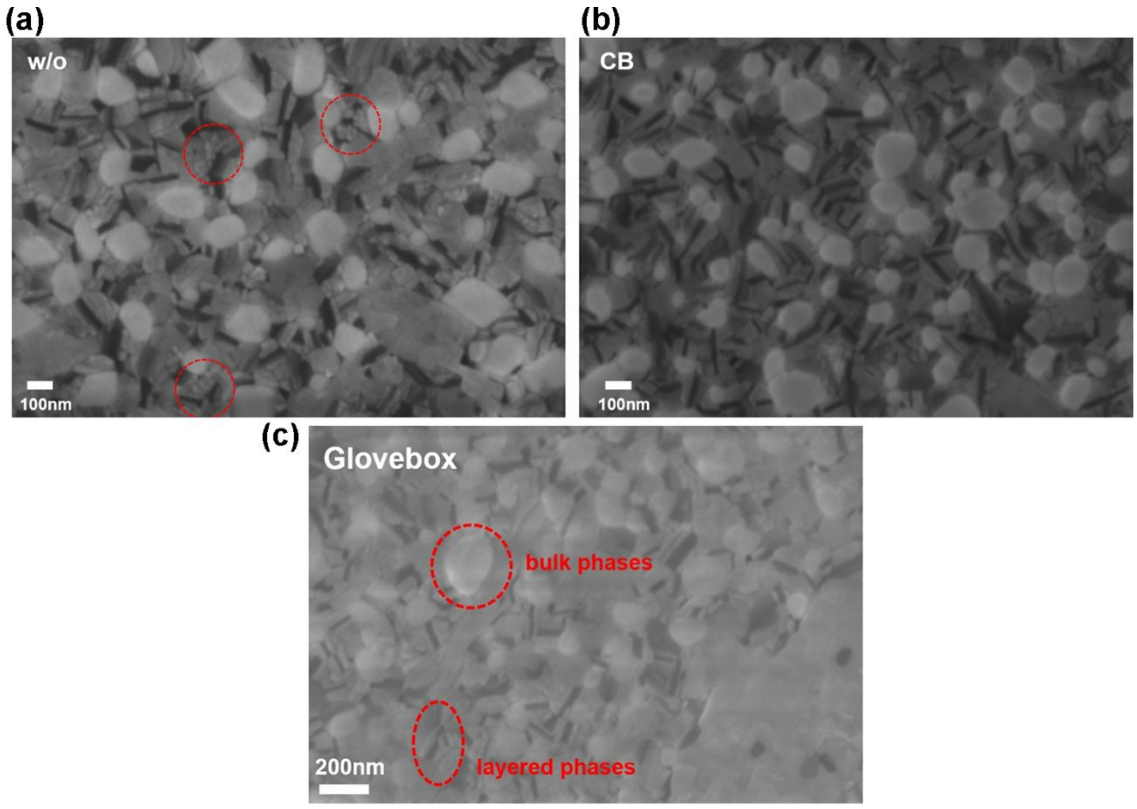

Figure S1. SEM images of perovskite films (a) without treatment, (b) treated under CB and (c) glovebox atmospheres.

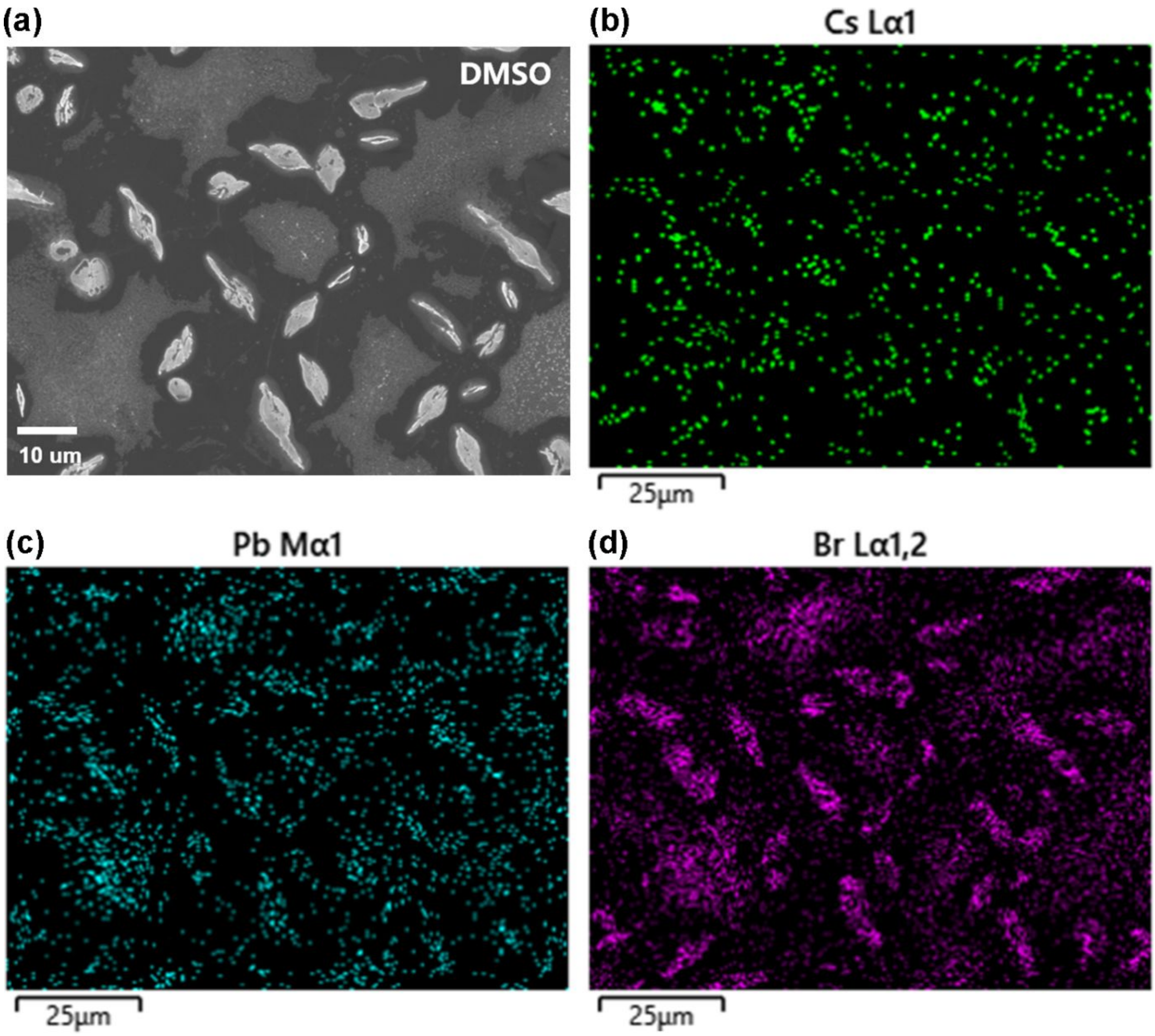

Figure S2. Morphological and component characterization of the perovskite films treated under DMSO solvent vapor. (a) Low magnification SEM image and (b-d) EDS mapping of the Cs, Pb and Br components.

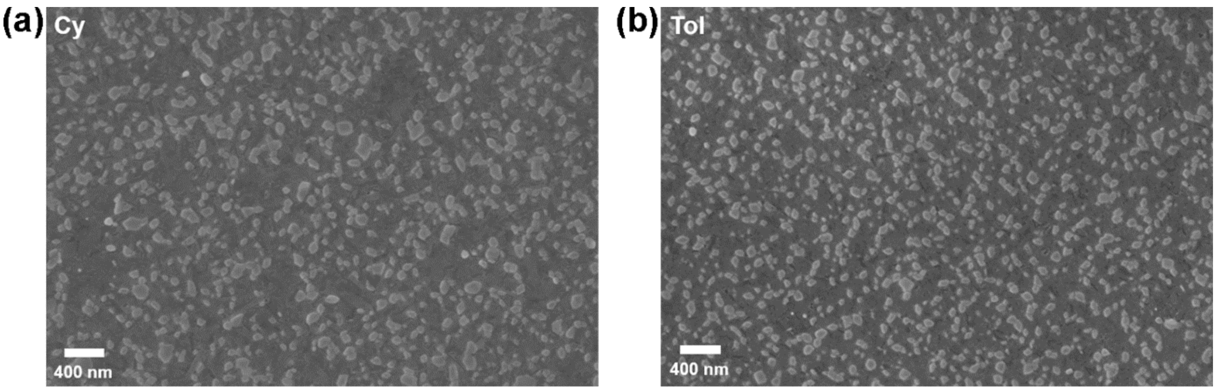

Figure S3. SEM images of perovskite films treated under non-polar solvent vapor (a) Cyclohexane and (b) Toluene.

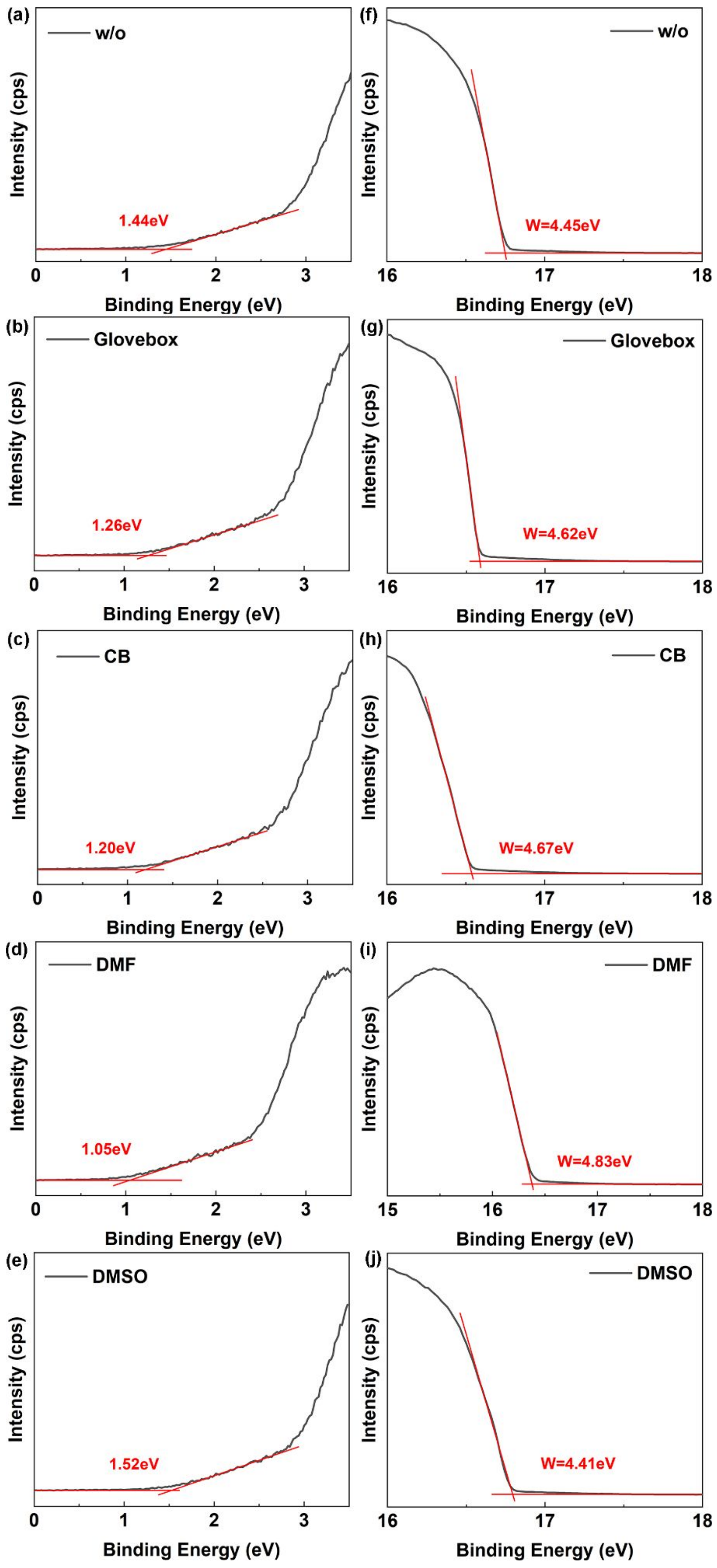

Figure S4. UPS spectra of (a-e) VB-edge regions and (f-j) high-binding energy secondary-electron cutoff of perovskite films treated under different atmospheres.

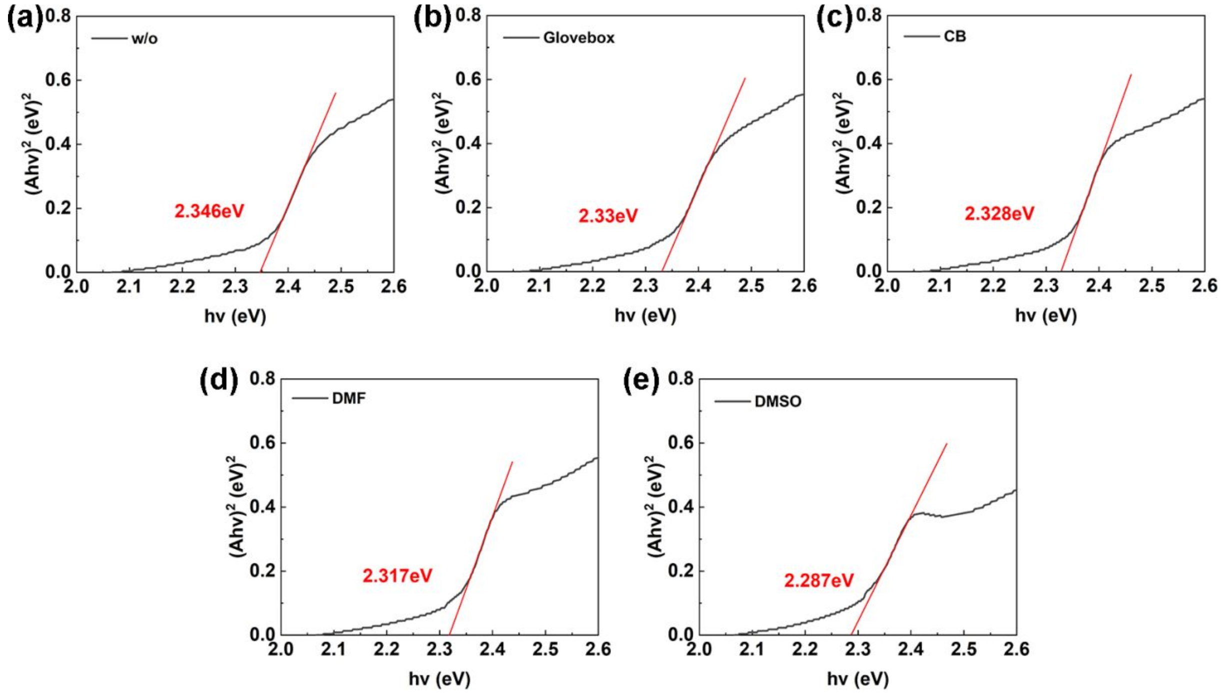

Figure S5. (a-e) Optical bandgaps of perovskite films extracted from absorption spectra.

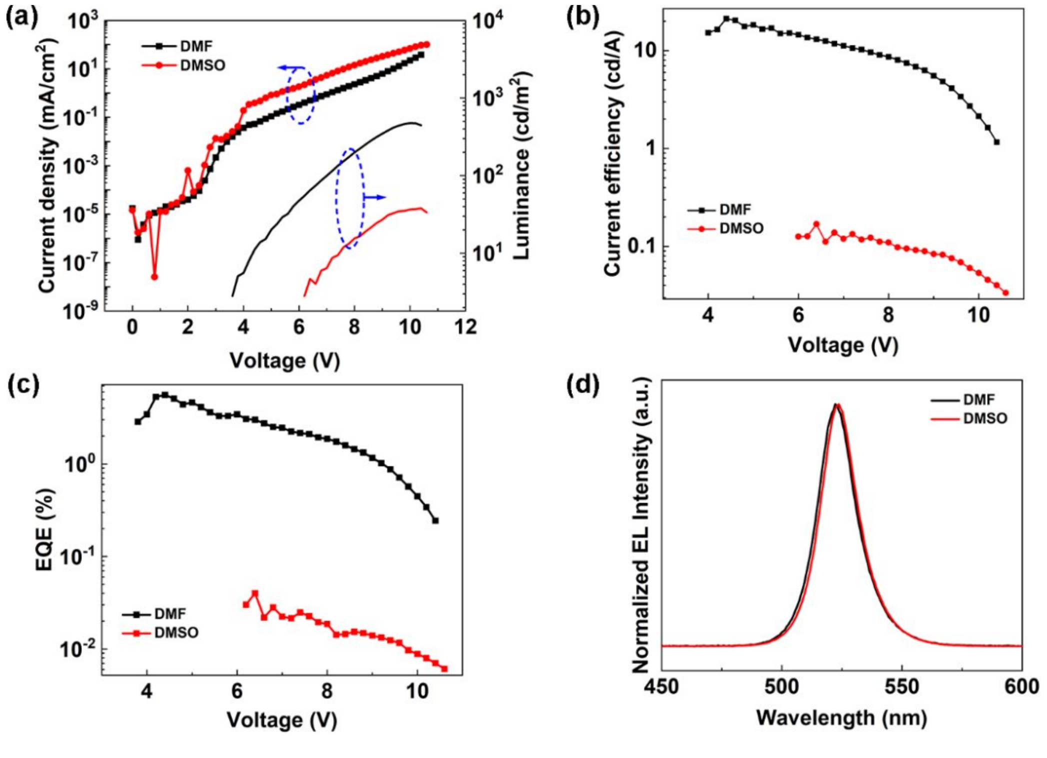

Figure S6. Device performances of PeLEDs treated under DMF and DMSO atmospheres. (a) J-L-V characteristics. (b) Current efficiency versus voltage curves. (c) EQE versus voltage curves. (d) EL spectra of PeLEDs under an applied voltage of 8V.

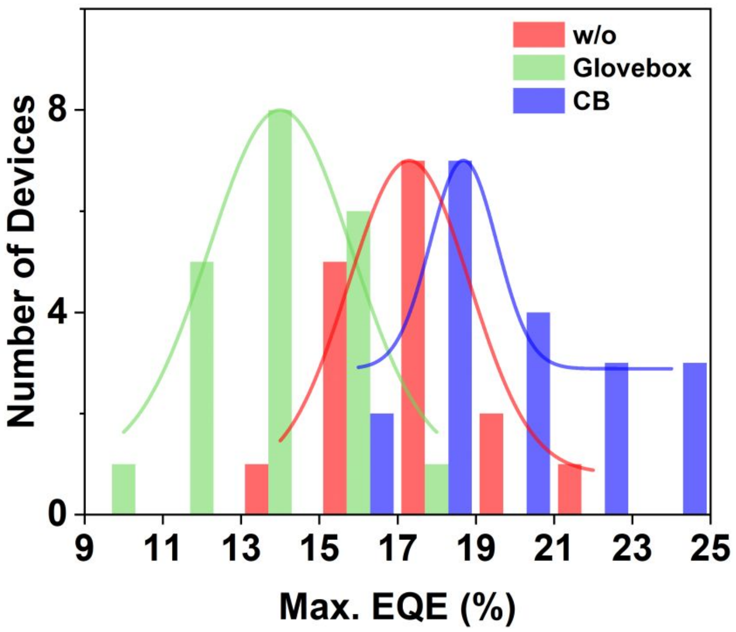

Figure S7. Histogram of peak EQEs of the devices without treatment, treated under glovebox and CB atmospheres.

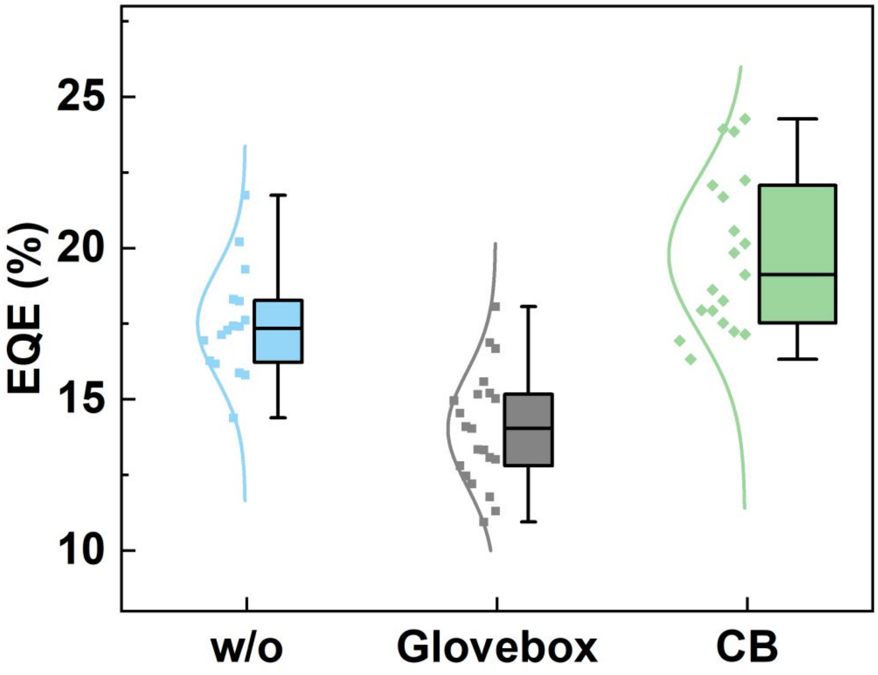

Figure S8. Box of EQE statistics of PeLEDs without treatment, treated under glovebox and CB atmospheres.

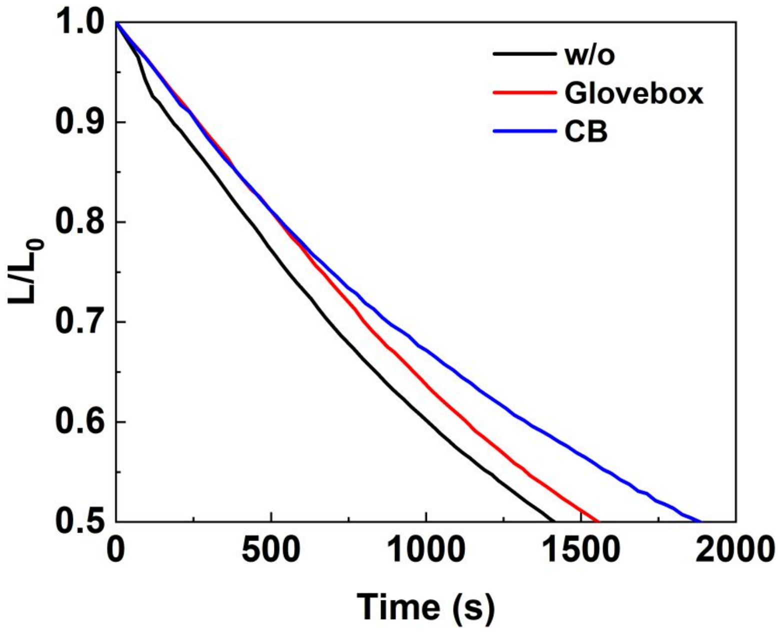

Figure S9. The operational stability of the devices without treatment, treated under glovebox and CB atmospheres, , each evaluated at an initial luminance of 100 cd m^{-2}.

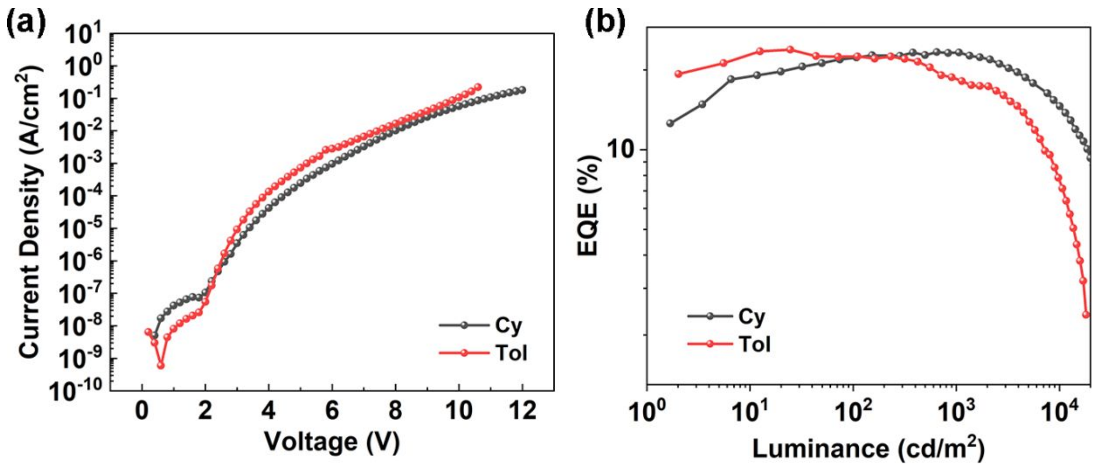

Figure S10. Device performances of PeLEDs treated under Cyclohexane (Cy) and Toluene (Tol) atmospheres. (a) J-V characteristics. (b) EQE versus luminance curves. The maximum EQEs reached 23.86% for toluene-treated and 23.36% for cyclohexane-treated devices, demonstrating the effectiveness of non-polar solvent vapor processing.
